# The yeast Hsp70 homolog Ssb: a chaperone for general de novo protein folding and a nanny for specific intrinsically disordered protein domains

**DOI:** 10.1007/s00294-016-0610-6

**Published:** 2016-05-26

**Authors:** Volker Hübscher, Kaivalya Mudholkar, Sabine Rospert

**Affiliations:** 1 Faculty of Medicine, ZBMZ, Institute of Biochemistry and Molecular Biology, University of Freiburg, 79104 Freiburg, Germany; 2 BIOSS Centre for Biological Signaling Studies, University of Freiburg, 79104 Freiburg, Germany

**Keywords:** *Saccharomyces cerevisiae*, 14-3-3, Bmh, Hsp70, Ssb, SNF1, AMPK, Glc7, Reg1, PKA

## Abstract

Activation of the heterotrimeric kinase SNF1 via phosphorylation of a specific residue within the α subunit is essential for the release from glucose repression in the yeast *Saccharomyces cerevisiae*. When glucose is available, SNF1 is maintained in the dephosphorylated, inactive state by the phosphatase Glc7-Reg1. Recent findings suggest that Bmh and Ssb combine their unique client-binding properties to interact with the regulatory region of the SNF1 α subunit and by that stabilize a conformation of SNF1, which is accessible for Glc7-Reg1-dependent dephosphorylation. Together, the 14-3-3 protein Bmh and the Hsp70 homolog Ssb comprise a novel chaperone module, which is required to maintain proper glucose repression in the yeast *S. cerevisiae*.

According to Anfinsen’s hypothesis any polypeptide folds into a defined 3D structure, which represents its energetically most favorable conformation (Anfinsen 1973). Many purified proteins can be denatured and subsequently refolded in vitro, demonstrating that their linear amino acid sequence provides all the information required for proper folding (Biter et al. 2016). However, there is an increasing number of polypeptides, which do not follow the ‘one polypeptide-one structure’ rule. Intrinsically disordered (ID) proteins, or ID domains within proteins, rather exist as a structural ensemble, either at the secondary or tertiary level (Uversky and Dunker 2010; Oldfield and Dunker 2014). ID proteins employ disorder to interact with multiple binding partners, which may induce different structures to the ID region (Uversky and Dunker 2010; Oldfield and Dunker 2014). It is now recognized that ID proteins are abundant, especially among eukaryotic phospho-proteins, which frequently contain disorder-promoting residues close to their phosphorylation sites (Bustos 2012). In general, hydrophilic residues, charged residues, and prolines are enriched within ID domains, while hydrophobic residues are mostly depleted (Hegyi and Tompa 2008; Oldfield and Dunker 2014). Some properties of ID proteins are similar to those of unfolded proteins, for example ID proteins are quickly degraded in vitro. However, in vivo half-lives of ID proteins do not differ from those of structured proteins (Hegyi and Tompa 2008; Oldfield and Dunker 2014). This led to the suggestion that ID domains might be protected from degradation via interaction with molecular chaperones (Oldfield and Dunker 2014), a heterogeneous group of proteins, which by definition interact with, and aid the folding or assembly of other proteins without being part of the final structures (Kim et al. 2013). However, extensive computational analysis of high-throughput interaction studies in different organisms, including yeast, revealed, that disorder in fact shows negative correlation with respect to chaperone binding (Hegyi and Tompa 2008). Based on the data it was suggested that ID proteins become functional without the aid of chaperones, and that those chaperones, which interact with ID proteins have evolved to perform specific functions rather than protecting ID proteins from aggregation or degradation (Hegyi and Tompa 2008).

When glucose is plentiful, a well-defined set of yeast genes, including many of those required for respiration, is turned off at the transcriptional level in a process termed glucose repression (Hedbacker and Carlson 2008; Smets et al. 2010; Conrad et al. 2014; Ho and Gasch 2015). When glucose becomes limiting, glucose repression is released and respiration is turned on. The major regulator of the release from glucose repression is the heterotrimeric kinase SNF1 (Hedbacker and Carlson 2008; Smets et al. 2010; Conrad et al. 2014) (Fig. 1a, b). SNF1 is highly conserved in eukaryotic organisms and its human homolog AMP-activated protein kinase (AMPK) is likewise activated to survive acute lack of energy or longer lean periods (Hedbacker and Carlson 2008; Conrad et al. 2014; Hardie 2014).

**Fig. 1:**
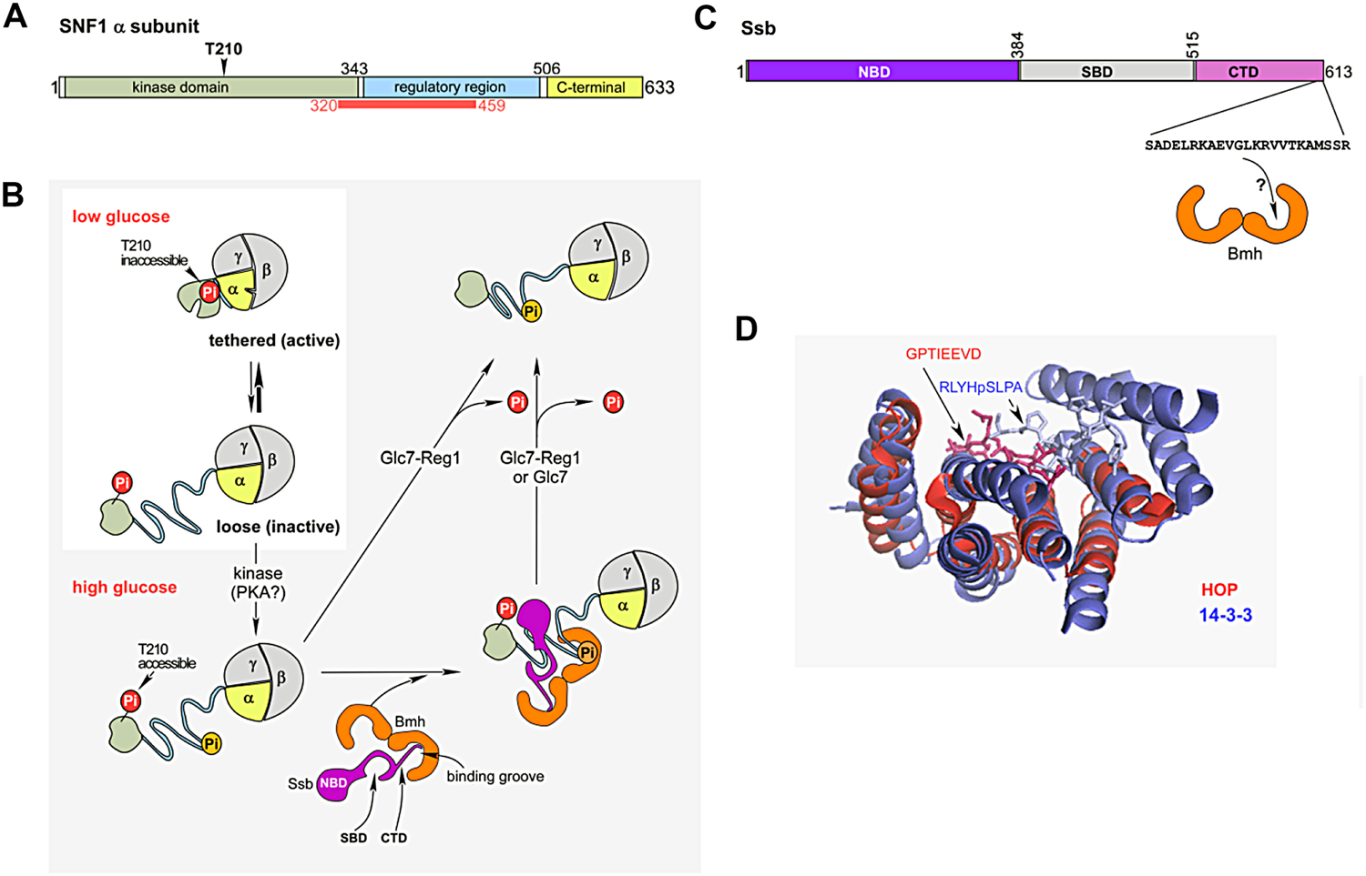
**a** Domain structure of the SNF1 α subunit. The kinase domain is indicated in beige, the regulatory region in blue, and the C-terminal domain in *yellow* (Chen et al. 2013; Xin et al. 2013; Hübscher et al. 2016). The region, which is predicted to be intrinsically disordered (Fukuchi et al. 2011) is indicated with the *red bar*. **b** Hypothetical role of Bmh/Ssb in the Glc7-dependent dephosphorylation of heterotrimeric SNF1. When glucose is low, SNF1 is phosphorylated at αT210 and the bulk of the kinase adopts the tethered active conformation, while only a minor fraction is in the loose, inactive conformation. When glucose becomes available a residue within one of the predicted 14-3-3 binding motifs of the α subunit may be phosphorylated (possibly by PKA, or another glucose-activated kinase), which would allow Bmh/Ssb to interact with SNF1. Bmh/Ssb then stabilizes the loose, inactive conformation and enhances the accessibility of αT210 for dephosphorylation by Glc7-Reg1. After the dephosphorylation of αT210 Bmh/Ssb is released from SNF1 (Hübscher et al. 2016). The *color code* of the SNF1 α subunit is as in **a**. The β and γ subunits are shown in *gray*. Phosphorylation of αT210 is indicated in *red*, phosphorylation of the potential residue involved in Bmh recruitment is shown in *orange*. Ssb is shown in *purple*, Bmh in *orange*. *NBD* nucleotide-binding domain, *SBD* substrate binding domain, *CTD* C-terminal domain of Ssb. The scheme incorporates previous models on the regulation of SNF1 activity (Chandrashekarappa et al. 2013; Conrad et al. 2014; Hübscher et al. 2016). For more details and references see text. **c** Domain structure of Ssb. *NBD* nucleotide-binding domain, *SBD* substrate binding domain, *CTD* C-terminal domain of Ssb. Shown is the amino acid sequence of the C-terminal residues of Ssb, which are required for the interaction between Bmh and Ssb in total cell extracts (Hübscher et al. 2016). If Ssb was bound to one binding groove of Bmh via its C-terminus, the other binding groove of Bmh plus the SBD of Ssb would be free to interact with client proteins (see **b**). **d** The interaction of the C-terminus of Hsc70 with its cochaperone HOP resembles the interaction of a client peptide with a 14-3-3 homolog. Superimposition of a 14-3-3 homolog in a complex with a phosphopeptide (1QJA, Rittinger et al. 1999) and of the TPR1 domain of HOP in complex with the C-terminal peptide of Hsc70 (1ELW, Scheufler et al. 2000). 14-3-3 and the phosphopeptide are shown in *blue*, the TRP1 domain of HOP, and the C-terminal Hsc70 peptide are shown in *red*

The catalytic subunit of the SNF1/AMPK heterotrimer is the α subunit, which is composed of an N-terminal kinase domain, followed by a regulatory region, and a C-terminal domain (Fig. 1a) (Hedbacker and Carlson 2008; Conrad et al. 2014). The C-terminal domain of the α subunit, together with the β and γ subunits form the so-called heterotrimeric core, to which the N-terminal kinase domain of the α subunit is more loosely connected (Chandrashekarappa et al. 2013; Chen et al. 2013; Xin et al. 2013; Conrad et al. 2014) (Fig. 1b). Interestingly, residues 320-459 of the α subunit, which cover most of the regulatory region, are predicted to be intrinsically disordered (DICHOT, Fukuchi et al. 2009). Crystal structures of AMPK revealed segments within the regulatory region, which transform from disordered to structured and vice versa depending on the activity status of the enzyme (Chandrashekarappa et al. 2013; Chen et al. 2013; Xin et al. 2013; Hardie 2014). SNF1/AMPK thus displays important hallmarks of an ID protein (Uversky and Dunker 2010).

Activation of SNF1/AMPK critically depends on two attributes. First, a single threonine residue within the kinase domain of the α subunit needs to be phosphorylated (Hedbacker and Carlson 2008; Conrad et al. 2014; Hardie 2014). In SNF1 the essential activating phosphorylation occurs on αT210 (Fig. 1a) (Hedbacker and Carlson 2008; Smets et al. 2010; Conrad et al. 2014). Second, SNF1 needs to adopt a conformation, in which the α subunit kinase domain is tethered to the heterotrimeric core complex (Chandrashekarappa et al. 2013; Conrad et al. 2014) (Fig. 1b). The upstream kinases responsible for the phosphorylation of αT210 are not regulated by the availability of glucose (Hedbacker and Carlson 2008; Rubenstein et al. 2008; Smets et al. 2010; Conrad et al. 2014). Instead, glucose regulates SNF1 activity at the level of αT210 dephosphorylation, which is mediated by the essential protein phosphatase Glc7 and its regulatory subunit Reg1 (Hedbacker and Carlson 2008; Smets et al. 2010; Conrad et al. 2014). While it is not understood how exactly the glucose signal transfers to SNF1, the available data fit to a model, in which SNF1 adopts a loose conformation with αT210 accessible for dephosphorylation when glucose is present, while most of SNF1 is tethered and αT210 is protected from dephosphorylation when glucose is absent (Fig. 1b) (Rubenstein et al. 2008; Chandrashekarappa et al. 2013; Conrad et al. 2014; Hübscher et al. 2016). Obviously, defects in glucose repression can result from a malfunction of the phosphatase system, as for example observed in a Δ*reg1* strain (Hedbacker and Carlson 2008; Conrad et al. 2014; Hübscher et al. 2016). However, the model also predicts that a shift of the equilibrium from the loose to the tethered conformation will result in hyperphosphorylation of SNF1 at αT210 and glucose repression defects. This is supported by the observation that mutations within SNF1, or drugs, which stabilize the tethered conformation protect αT210 from dephosphorylation (Chandrashekarappa et al. 2013).

Proper glucose repression depends on the 14-3-3 homolog Bmh and the Hsp70 homolog Ssb, and both, Bmh and Ssb, directly interact with SNF1, Glc7, and Reg1 (Bruckmann et al. 2004, 2007; Dombek et al. 2004; Ichimura et al. 2004; Elbing et al. 2006; Kakiuchi et al. 2007; van Heusden 2009; von Plehwe et al. 2009; Panni et al. 2011; Hübscher et al. 2016). 14-3-3 proteins are highly conserved in eukaryotic cells and form either homo- or heterodimers. Each of the subunits contains an amphipathic groove that binds to short, phosphorylated, consensus motifs within client proteins. The ability to distinguish between phosphorylated and dephosphorylated consensus motifs is essential for the role of 14-3-3 proteins in the regulation of processes like signaling, transcription, intracellular localization, and enzymatic catalysis (Obsil and Obsilova, 2011; Bustos 2012). By employing both of its binding grooves a single heterodimeric 14-3-3 protein can induce conformational changes, mask regions, or facilitate the interaction between two client proteins (Obsil and Obsilova 2011). Of note, 14-3-3 consensus motifs are most frequently located within hydrophilic and highly charged ID domains (Hegyi and Tompa 2008; Bustos 2012; Oldfield and Dunker 2014). In contrast, Hsp70 chaperones, via their substrate binding domain (SBD), interact preferentially with extended stretches of hydrophobic sequences. The affinity of Hsp70s for substrates is regulated allosterically by nucleotide binding to their nucleotide-binding domain (NBD) (Fig. 1c) (Peisker et al. 2010; Kim et al. 2013; Clerico et al. 2015).

Ssb is one of 86 predicted high-confidence interaction partners of Bmh (Panni et al. 2011) and crosslinking experiments indicate that Bmh and Ssb contact each other directly (Hübscher et al. 2016). The interaction of Ssb with Bmh in total cell extracts depends on the very C-terminus of Ssb (Hübscher et al. 2016), suggesting that Ssb may bind to Bmh in a client protein-like manner (Fig. 1c, d). With respect to the interaction between Ssb and Bmh there is interesting similarities to another chaperone/cochaperone couple. The very C-terminus of Ssa-type Hsp70 homologs is comprised of the residues EEVD. Hsp70 homologs, which contain the EEVD tail can interact with tetratricopeptide (TPR) domain cochaperone partners (Scheufler et al. 2000; Clerico et al. 2015). For example, mammalian HOP and its yeast homolog Sti1 interact with the EEVD motif via their TPR domains (Scheufler et al. 2000; Röhl et al. 2015). Interestingly, the fold of the TPR domain is structurally related to the fold of 14-3-3 homologs (Das et al. 1998). Indeed it was noted that an EEVD-peptide bound to TPR1 of HOP superpositions with respect to functionally important residues with a phospho-peptide bound to a 14-3-3 protein (Scheufler et al. 2000) (Fig. 1d). The findings suggest that the interaction of Hsp70s with either HOP/Sti1, or with 14-3-3 is determined by the very C-terminus of the respective Hsp70, but follows the same architectural principles. Interestingly, the interaction between Hsp70s and TPR domains in some cases is regulated via phosphorylation close to the EEVD motif (Assimon et al. 2015). Exactly this type of regulation is expected for the interaction between Ssb and Bmh. Of note, the observation that a 14-3-3 protein functionally interacts with Hsp70 is not without precedence. In the so-called guidance complex a plant 14-3-3 homolog together with an Hsp70 enhances the import efficiency of certain preproteins into chloroplasts in vitro (Flores-Perez and Jarvis 2013). Recently it was discovered that a mammalian 14-3-3 homolog, phosphorylated BAG3, and Hsp70 interact and play a role in the aggresome-macroautophagy pathway (Jia et al. 2014).

One important observation which sheds light on the mechanism by which Bmh/Ssb affect glucose repression is the strong multicopy suppressor effect of Bmh and Ssb with respect to SNF1 αT210 hyperphosphorylation in a Δ*reg1* strain (von Plehwe et al. 2009; Hübscher et al. 2016). The findings indicate that increased cellular levels of Bmh, or Ssb, allow Glc7 to dephosphorylate αT210 even in the absence of Reg1 (Hübscher et al. 2016). This fits to a model in which Bmh and Ssb bind to, and by that stabilize, the loose conformation of SNF1 (Fig. 1b) (Hübscher et al. 2016). ID domains bind to partner proteins with high specificity but low affinity (Bustos 2012). Thus, increasing the cellular concentration of the Bmh/Ssb complex may indeed enhance the fraction of SNF1 in a complex with Bmh/Ssb by the law of mass action. Interestingly, αT210 phosphorylation is strictly required for the binding of Bmh to SNF1 (Hübscher et al. 2016). Because αT210 is not situated in a 14-3-3 consensus motif, it is unlikely that this residue establishes the critical, direct contact to Bmh. However, a 14-3-3 binding motif, hidden in the tethered SNF1 complex, may become accessible in the loose conformation, first for phosphorylation and subsequently for binding of Bmh (Fig. 1b) (Hübscher et al. 2016).

SNF1 contains a number of phosphorylated Ser/Thr residues, aside from αT210 (Stark et al. 2010) and also contains a number of potential 14-3-3 binding sites (Panni et al. 2011). Upstream kinases responsible for SNF1 phosphorylation at residues other than αT210, are currently not known. One potential candidate is PKA, not least because recent observations suggest that glucose-mediated activation of the PKA pathway is involved in αT210 dephosphorylation (Conrad et al. 2014). To that end, active PKA enhances the activity of Glc7-Reg1 (Castermans et al. 2012; Conrad et al. 2014). However, active PKA may also further the dephosphorylation of αT210 via promoting—directly or indirectly—the phosphorylation of SNF1 residues, required for the recruitment of Bmh/Ssb.

While the role of Bmh in glucose repression is in line with the expected function of the 14-3-3 homolog (van Heusden 2009), the role of Ssb in this process is rather unexpected. Ssb can bind to cytosolic ribosomes directly and then interacts with more than 3000 unfolded polypeptide chains emerging from the ribosomal polypeptide exit tunnel (Gong et al. 2009; Peisker et al. 2010; Willmund et al. 2013). With this function in mind, one may speculate that Ssb, when recruited to SNF1 via Bmh, may interact with intrinsically disordered segments of the α subunit regulatory domain (Fig. 1a). Interestingly, it was reported that nascent chains, which interact with Ssb are enriched for ID proteins (Willmund et al. 2013). In a way, these observations are surprising, because the amino acid composition of ID domains does not resemble those of Hsp70 substrates (Hegyi and Tompa 2008; Kim et al. 2013; Oldfield and Dunker 2014; Clerico et al. 2015). Possibly, the SBD of Ssb displays some special properties different from other Hsp70 homologs. Alternatively, Bmh may cooperate with Ssb in regulatory processes precisely because the Hsp70 homolog possesses a substrate range distinct from Bmh itself. While Bmh may bind to an ID domain, Ssb may interact with adjacent, more hydrophobic regions of a client. Of note, the very specific effect exerted by Bmh/Ssb on the dephosphorylation of SNF1 αT210, without affecting the expression level of SNF1, or Glc7-Reg1 (von Plehwe et al. 2009; Hübscher et al. 2016) indicates that Ssb is not required to chaperone SNF1 or Glc7-Reg1. Rather Ssb may act as a nanny, which reversibly interacts with the purpose* not to *permanently affect disorder within the client (Tsvetkov et al. 2009). As pointed out previously, this is the opposite of what canonical chaperones are supposed to do (Tsvetkov et al. 2009). Ssb thus seemingly serves a dual function in the cell. First, Ssb acts as a chaperone, which promotes the folding of numerous structured proteins, and second, Ssb acts as a nanny (Tsvetkov et al. 2009) keeping an eye on specific protégés, however, being more tolerant than a chaperone when it comes to promiscuous interactions.
